# Prevalence of suicide attempts in bipolar disorder: a systematic review and meta-analysis of observational studies

**DOI:** 10.1017/S2045796019000593

**Published:** 2019-10-25

**Authors:** Min Dong, Li Lu, Ling Zhang, Qinge Zhang, Gabor S. Ungvari, Chee H. Ng, Zhen Yuan, Yifan Xiang, Gang Wang, Yu-Tao Xiang

**Affiliations:** 1 Guangdong Mental Health Center, Guangdong Provincial People's Hospital, Guangdong Academy of Medical Sciences, Guangzhou, China; 2 Unit of Psychiatry, Institute of Translational Medicine, Faculty of Health Sciences, University of Macau, Macao SAR, China; 3 The National Clinical Research Center for Mental Disorders & Beijing Key Laboratory of Mental Disorders, Beijing Anding Hospital & the Advanced Innovation Center for Human Brain Protection, Capital Medical University, School of Mental Health, Beijing, China; 4 Division of Psychiatry, School of Medicine, University of Western Australia, Perth, Australia; 5 University of Notre Dame Australia, Fremantle, Australia; 6 Department of Psychiatry, The Melbourne Clinic and St Vincent's Hospital, University of Melbourne, Richmond, Victoria, Australia; 7 Pui Ching Middle School Macau, Macau SAR, China; 8 Center for Cognition and Brain Sciences, University of Macau, Macao SAR, China

**Keywords:** Bipolar disorder, meta-analysis, prevalence, suicide attempt

## Abstract

**Aims:**

Bipolar disorder (BD) is a severe psychiatric disorder associated with a high risk of suicide. This meta-analysis examined the prevalence of suicide attempts (SA) in patients with BD and its associated factors.

**Methods:**

A systematic literature search was conducted in the PubMed, PsycINFO, EMBASE and Web of Science databases from their inception to 11 June 2018. The prevalence of SA in BD was synthesised using the random-effects model.

**Results:**

The search identified 3451 articles of which 79 studies with 33 719 subjects met the study entry criteria. The lifetime prevalence of SA was 33.9% (95% CI 31.3–36.6%; *I*^2^ = 96.4%). Subgroup and meta-regression analyses revealed that the lifetime prevalence of SA was positively associated with female gender, BD-I, BD Not Otherwise Specified and rapid cycling BD subtypes, income level and geographic region.

**Conclusion:**

This meta-analysis confirmed that SA is common in BD and identified a number of factors related to SA. Further efforts are necessary to facilitate the identification and prevention of SA in BD. Long-term use of mood stabilisers coupled with psycho-social interventions should be available to BD patients to reduce the risk of suicidal behaviour.

## Introduction

Bipolar disorder (BD) is a mood disorder characterised by recurrent depressive and manic/hypomanic episodes (Goodwin and Jamison, 2007), with different subtypes, such as bipolar I (BD-I), bipolar II (BD-II) and BD Not Otherwise Specified (BD-NOS) (American Psychiatric Association, 2013). The lifetime prevalence of BD is estimated to be 2.4% worldwide (Merikangas *et al*., 2011). Due to its complex and varying clinical presentations, BD is often misdiagnosed for other psychiatric disorders (Berk *et al*., 2007), such as major depression (Perlis, 2005) and substance abuse disorder (Patel *et al*., 2015), which results in poor treatment outcomes and increased risk of suicide (Rosa *et al*., 2010).

Suicide attempt (SA) is an important predictor of completed suicide (Drake *et al*., 1985; Harkavy-Friedman *et al*., 1999; Kessler *et al*., 2005). Compared to the general population, BD patients are at a higher risk of suicide (Nierenberg *et al*., 2001; Plans *et al*., 2019). A prospective study found that BD had the highest risk of suicide of all psychiatric diagnoses (Brown *et al*., 2000). The risk of SA in BD is approximately 30-fold higher than that in the general population (Goodwin and Jamison, 2007), and approximately 0.9% of BD patients attempt suicide every year (Beyer and Weisler, 2016). Further, the combination of BD and history of SA is probably the strongest predictor of completed suicide (Antypa *et al*., 2013). Approximately 30–50% of BD patients have a lifetime history of SA, of whom 15–20% eventually died by suicide (Baldessarini *et al*., 2006; Gonda *et al*., 2012). In a previous study, more lethal methods of SA and suicide were observed in BD patients than in the general population (Simon *et al*., 2007). Thus, suicide and SA account for a considerable proportion of disease burden in BD (Angst, 2004).

A host of demographic and clinical factors are related to SA in BD, including female gender, single and divorced marital status, history of sexual abuse, younger age of onset, depressive phase, severe depressive symptoms, frequent hospitalisation for depression, BD-I subtype, rapid cycling, comorbid substance abuse and other psychiatric disorders, past history of SA and suicide in first-degree relatives (Leverich *et al*., 2003; Hawton *et al*., 2005; Shabani *et al*., 2013; Schaffer *et al*., 2015*a*, 2015*b*; Bobo *et al*., 2018). Epidemiological studies on the prevalence of SA in BD are fraught with methodological limitations resulting in inconsistent findings (Novick *et al*., 2010; Latalova *et al*., 2014; Tondo *et al*., 2016). In previous reviews, relying on only one database (PsycINFO) (Novick *et al*., 2010) or inclusion of randomised control trials with stringent entry criteria are just two examples of methodological shortcomings that limit the generalisability of the findings (Tondo *et al*., 2016).

In order to inform health care policy and frontline mental health professionals in their attempt to reduce suicide risk, it is important to understand the patterns of SA and their related factors. In view of the large number of recently published epidemiological studies of SA in BD (Passos *et al*., 2016; Baldessarini *et al*., 2017; Bellivier *et al*., 2017; Bezerra *et al*., 2017; Cremaschi *et al*., 2017; Kattimani *et al*., 2017; Altamura *et al*., 2018; Bobo *et al*., 2018; Duko and Ayano, 2018), this systematic review and meta-analysis explored the prevalence of SA in BD and its associated factors. The main hypothesis of the study was that bipolar patients would have a significantly higher prevalence of SA compared to the general population. In order to make the sample more representative, only epidemiological studies were included in this meta-analysis.

## Methods

### Search strategy

**Fig. 1.**
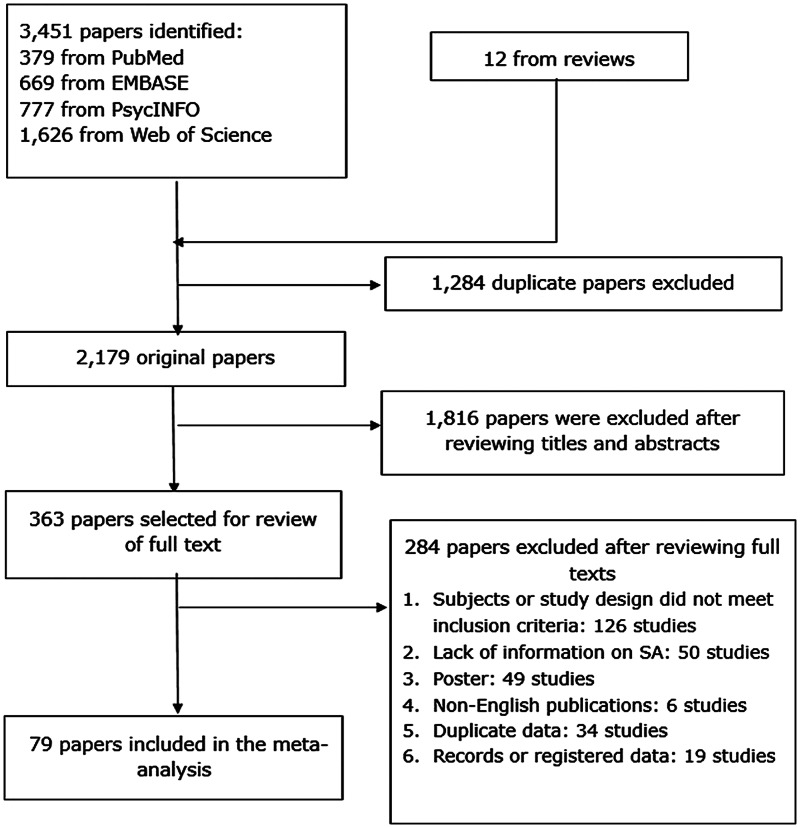
Flowchart of study selection.

This meta-analysis followed the Preferred Reporting Items for Systematic Review and Meta-Analyses (PRISMA) and MOOSE recommendations (Stroup *et al*., 2000). The registration number of this protocol in the International Prospective Register of Systematic Reviews (PROSPERO) is CRD42018108290. Two authors (MD and LL) searched the PubMed, PsycINFO, EMBASE and Web of Science databases from their inception through 11 June 2018 using the following search terms: attempted suicide, suicide attempt*, bipolar disorder, manic-depressive disorder, affective disorder, mood disorder, bipolar depression, bipolar affective, hypomania, mania, manic, epidemiology, cross-sectional study, cohort study, observational study, prevalence, rate, percentage, proportion. References of review papers were also searched to identify additional articles. Figure 1 displays the process of the selection of studies for the meta-analysis.

### Study selection

Two authors (MD and LL) independently screened the title and abstract of relevant studies and then read their full text for eligibility. Inclusion criteria were: (1) BD diagnosed according to any international or local diagnostic criteria; (2) available meta-analysable data on SA with one or more of the following timeframes: lifetime, 1-year, 1-month, current episode and from illness onset; (3) cross-sectional or cohort studies (only baseline data of cohort studies were analysed); (4) publication in English. Studies were excluded if they (1) were intervention studies or reviews; (2) had very small sample size (*n* < 100) (Walker *et al*., 2013; Matte *et al*., 2016) or special populations, such as adolescent or elderly samples; (3) were registry-based studies or extracted data from medical records. If multiple papers were published based on the same dataset, only the study with the largest sample size was analysed. Several papers (such as Carballo *et al*., 2008; Elizabeth Sublette *et al*., 2009; Studart *et al*., 2016) reported overlapping data with the included studies. The corresponding authors were contacted for clarification, but since no reply was received, therefore these studies were excluded. Any uncertainty in the literature search and study selection was resolved by a discussion with a senior researcher (YTX).

### Data extraction and quality assessment

Demographic and clinical data were extracted from the included studies covering the first author, publication year, time of survey, study site, sampling method, study design, sample size, mean age, proportion of males, diagnostic criteria of BD, patient source, assessment and timeframe of SA. Data extraction was performed independently by two authors (MD and LL).

Study quality was assessed using the quality assessment instrument for epidemiological studies (Boyle, 1998; Loney *et al*., 1998); which has the total score ranging from 1 (lowest quality) to 8 (highest quality) points. The eight domains of the instrument are: (1) Target population is clearly defined. (2) Probability or entire population sampling is used. (3) Response rate is >70%. (4) Non-responders are clearly described. (5) Sample is representative of the target population. (6) Data collection methods are standardised. (7) Validated criteria are used to assess the presence of disease. (8) Estimates of prevalence are given with confidence intervals and detailed by subgroup (if applicable). Studies with a total score of 7–8 were considered as high quality, 4–6 as moderate quality and 0–3 as low quality (Yang *et al*., 2016).

### Statistical analysis

Data analyses were performed with the Comprehensive Meta-Analysis (CMA), Version 2.0 (Biostat Inc., Englewood, NJ, USA) and STATA, Version 12.0 (Stata Corporation, College Station, TX, USA). A random-effect model was applied to calculate the overall prevalence and its 95% confidence interval (95% CI). Heterogeneity between studies was assessed with the *I*^2^ statistic. Sensitivity analysis was performed by excluding studies one by one to explore the impact of each study on the overall results. Publication bias was evaluated with funnel plots and the Begg regression asymmetry test. In order to examine the moderating effects caused by demographic and clinical variables on the results, subgroup analyses for categorical variables (rapid cycling, gender, source of patients, income level, region and study design) and meta-regression analyses for continuous variables (per cent of BD subtype, sample size, mean age, illness duration and age of onset) were performed. If the number of studies was <10, subgroup analyses were conducted for continuous variables, using the median splitting method (Higgins and Green, 2008). All the tests were two-tailed, and *p* < 0.05 was considered significant.

## Results

The search initially identified 3451 articles from the target databases and 12 additional articles from other sources (Fig. 1). Altogether, 79 studies covering 33 719 patients met study entry criteria (online Supplementary Table S1), including 58 cross-sectional and 21 cohort studies published between 1985 and 2018. Twenty, six and four studies employed consecutive, convenience and random sampling, respectively. In terms of diagnosis, 71 and four studies applied the DSM and Research Diagnostic Criteria (RDC) (Spitzer *et al*., 1978), respectively, and one study each used the ICD, the DSM or/and ICD, DSM or/and RDC, the Affective Disorder Evaluation (ADE) (Sachs *et al*., 2003). The mean age of the whole sample ranged between 29.0 and 55.8 years, and then proportion of males ranged from 23.3 to 72.1%. Seventy-five of the 79 studies reported lifetime prevalence, three studies reported 1-year prevalence and three reported current episode prevalence of SA. The total score of quality assessment ranged from 4 to 8, with six studies rated as of high quality, and 73 studies as of moderate quality (online Supplementary Table S3).

### Pooled prevalence of SA

**Fig. 2.**
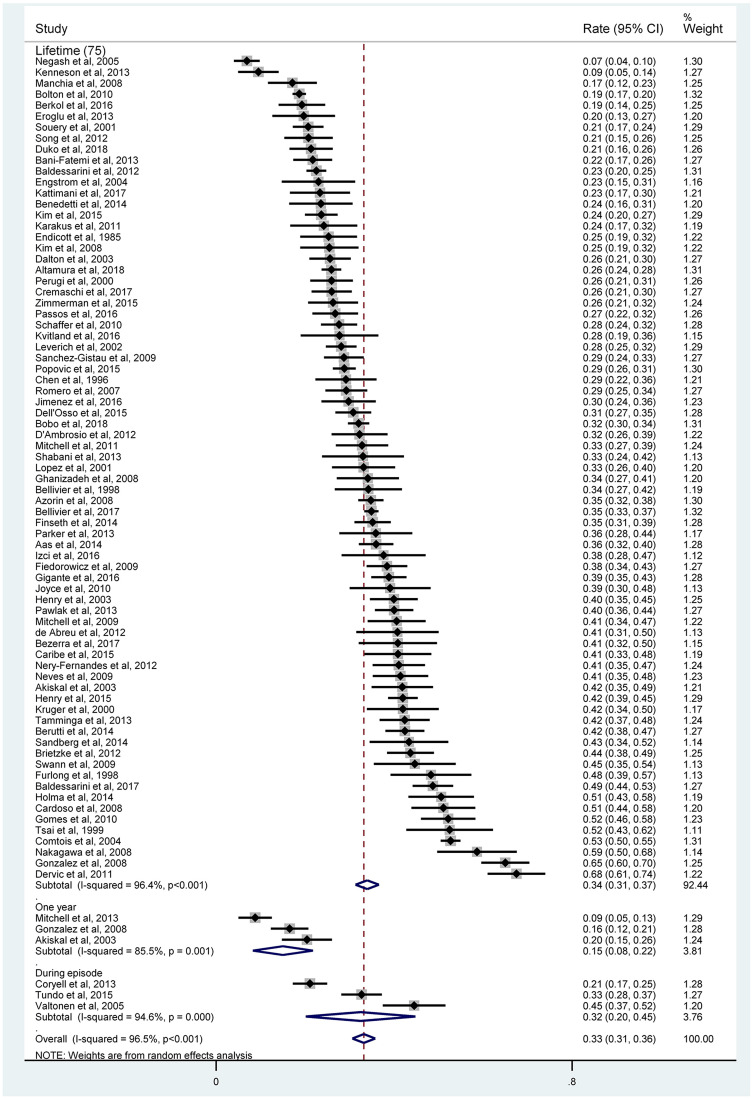
Forest plot of suicide attempt.

The pooled lifetime prevalence of SA in BD was 33.9% (95% CI 31.3–36.6%; *I*^2^ = 96.4%), 1-year prevalence of SA was 15.0% (95% CI 8.2–21.8%; *I*^2^ = 85.5%) and current episode prevalence of SA was 32.5% (95% CI 20.1–44.8%; *I*^2^ = 94.6%) (Fig. 2). None of the included studies reported 1-month prevalence or prevalence from illness onset.

### Sensitivity analysis and publication bias

When sensitivity analysis omitted each study one by one, there was no outlying study that could have significantly changed the lifetime prevalence of SA (online Supplementary Fig. S1). Neither the funnel plot nor Begg's test found significant publication bias with respect to the lifetime prevalence of SA (*z*  =  0.76, *p*  =  0.44) (online Supplementary Fig. S2).

### Subgroup and meta-regression analyses

The results of subgroup analyses are shown in Table 1. Gender, income level, rapid cycling and region were significantly associated with lifetime prevalence of SA, while study design was significantly associated with its 1-year prevalence. Specifically, the lifetime prevalence of SA in rapid cycling BD (47.0%) was significantly higher than in the non-rapid cycling subtype (30.2%) (*p* < 0.001). Female gender (34.4%) was significantly more prevalent than male gender (26.4%) (*p*  =  0.002) in SA in BD. The prevalence of SA in high- (33.7%) and middle-income countries (34.7%) was significantly higher than in low-income countries (12.8%) (*p*  =  0.003). The prevalence of SA was highest in the Americas (37.0%) and lowest in Africa (12.8%) (*p*  =  0.01). The 1-year prevalence of SA reported in cohort studies (18.2%) was significantly higher than in cross-sectional studies (8.8%) (*p*  =  0.003).

**Table 1.** Subgroup analyses of SA prevalence in bipolar disorder

Subgroup	Categories (No. of studies)	Patients with SA	Total patients with BD	Prevalence (%)	95% CI (%)	*I*^2^ (%)	*p* values within subgroup^a^	*Q (p* values across subgroups)^b^
*Lifetime prevalence of SA*								
	Total (75)	10 790	32 477	**33**.**9**	31.3–36.6	96.4	<0.001	
RC/NRC	RC (9)	713	1668	**47.0**	40.7–53.4	72.9	<0.001	17.83 (**<0.001**)
	NRC (9)	1521	4900	**30.2**	25.9–34.9	86.9	<0.001	
Gender	Male (25)	1059	4022	**26.4**	23.1–30.0	80.1	<0.001	9.21 **(0.002)**
	Female (25)	1984	5462	**34.4**	30.7–38.2	83.3	<0.001	
Source of patients	Inpatient (9)	1989	4515	**38.3**	32.2–44.8	94.3	<0.001	2.08 (0.35)
	Outpatient (26)	2571	7542	**33.0**	29.6–36.6	91.7	<0.001	
	Mix (17)	2793	8435	**34.1**	29.9–38.6	89.6	<0.001	
Income	High (52)	8375	24 941	**33.7**	30.8–36.8	95.6	<0.001	11.61 (**0.003**)
	Middle (16)	1598	4744	**34.7**	29.4–40.5	93.3	<0.001	
	Low (2)	78	562	**12.8**	6.7–23.1	95.5	<0.001	
Region	Africa (2)	78	562	**12.8**	6.7–23.2	95.5	<0.001	14.56 (**0.01**)
	The Americas (24)	4317	12 207	**37.0**	32.4–41.7	97.5	<0.001	
	Eastern Mediterranean (2)	93	276	**33.6**	19.8–50.9	0	0.85	
	Europe (32)	4627	13 864	**32.5**	28.8–36.5	90.1	<0.001	
	South-East Asia (1)	35	150	**23.3**	9.9–45.8	–	–	
	Western Pacific (8)	519	1696	**33**.**0**	25.7–41.2	88.3	<0.001	
Study design	Cross-sectional (56)	6883	21 185	**32.3**	29.4–35.3	95.5	<0.001	1.63 (0.20)
	Cohort (19)	3826	11 292	**36.1**	31.1–41.6	94.6	<0.001	
*One-year prevalence of SA*								
	Total (3)	108	710	**15.0**	8.2–21.8	85.5	<0.001	
Sample size^c^	>217 (1)	49	297	**16.5**	4.9–42.8	–	–	0.067 (0.796)
	⩽217 (2)	59	413	**13.7**	5.7–29.4	90.7	0.001	
Study design	Cross-sectional (1)	19	217	**8.8**	5.5–13.6	–	–	8.74 **(0.003)**
	Cohort (2)	89	493	**18.2**	14.7–22.3	17.7	0.27	
*Prevalence of SA during episode*								
	Total (3)	308	1025	**32.5**	20.1–44.8	94.6	<0.001	
Sample size^c^	<407 (1)	85	191	**44.5**	25.6–65.2	–	–	2.30 (0.129)
	⩾407 (2)	223	834	**26.5**	16.7–39.3	92.9	<0.001	
Study design	Cross-sectional (1)	133	407	**32.7**	9.6–68.8	–	–	0.003 (0.95)
	Cohort (2)	175	618	**31.6**	13.6-57.5	97.0	<0.001	

BD, bipolar disorder; NOS, Not Otherwise Specified; NRC, non-rapid cycling; RC, rapid cycling; SA, suicide attempt.

a Test of heterogeneity within subgroups.

b Test of prevalence of SA across subgroups.

c Median splitting method was used.

Bold values  =  *p* < 0.05.

In meta-regression analyses, the proportions of BD-I (*B*  =  0.002, *z*  =  3.9, *p* < 0.001) and BD-NOS (*B*  =  0.01, *z*  =  6.3, *p* < 0.001) were positively, while sample size (*B* = −0.00004, *z* = −3.5, *p* < 0.001) and the proportion of BD-II (*B* = −0.003, *z* = −4.9, *p* < 0.001) were negatively associated with lifetime prevalence of SA. Mean age (*B* = −0.004, *z* = −1.4, *p*  =  0.13), illness duration (*B* = −0.004, *z* = −0.8, *p*  =  0.40) and age of onset (*B* = −0.004, *z* = −0.7, *p*  =  0.46) were not significantly associated with lifetime prevalence of SA.

## Discussion

In this meta-analysis, the lifetime prevalence of SA in BD (33.9%; 95% CI 31.3–36.6%) was significantly higher than in the general population (0.8%; 95% CI 0.7–0.9%) (Bernal *et al*., 2007; Cao *et al*., 2015) and in schizophrenia (14.6%; 95% CI 9.1–22.8%), but was only slightly higher than the figures found in major depression (31%; 95% CI 27–34%) (Howe *et al*., 2014; Dong *et al*., 2017, 2018). The higher risk of SA in BD compared to major depression relates to the differences in clinical profile between both disorders (Szadoczky *et al*., 2000). Impulsivity, a trait of BD (Tondo and Baldessarini, 2005), is associated with both SA and suicide (Swann *et al*., 2005). In addition, several key factors contributing to SA, e.g. mixed state and depressive phase, are part of BD (Oquendo *et al*., 2000; Schaffer *et al*., 2015*a*). However, the difference between BD and depression with regard to lifetime prevalence of SA did not reach a significant level (33.9%, 95% CI 31.3–36.6% *v*. 31%, 95% CI 27–34%) in a meta-analysis (Dong *et al*., 2018). Another review of 24 studies (Novick *et al*., 2010) reported the lifetime prevalence of SA (36.3% in BD-I and 32.4% in BD-II). However, in the Novick *et al*. review, only the PsycINFO database was searched yielding only 439 relevant hits and the prevalence estimates of SA in different timeframes were not reported. In contrast, the current meta-analysis more comprehensively covered the literature and examined the prevalence of SA in different timeframes. Novick *et al*. analysed randomised control trials with stringent entry criteria, which could lead to selection bias, while this meta-analysis included observational studies, thereby increasing the representativeness of the study sample. Furthermore, more sophisticated analyses including subgroup and sensitivity analyses were carried out in the present study.

The lifetime prevalence of SA was higher in females than in males, which is also found in previous studies (Tondo *et al*., 2003, 2016). The possible reasons include relatively more frequent depressive episodes (Schneck *et al*., 2004), rapid cycling BD (Cruz *et al*., 2008) and history of childhood physical and sexual abuse (Stefanello *et al*., 2008) in female patients. Male BD patients are more likely to resort to more lethal methods of suicide (Pompili *et al*., 2009). Previous findings comparing the SA rate between BD-I and BD-II have been inconsistent. Higher SA rates in BD-I (Oquendo *et al*., 2010; Antypa *et al*., 2013; Bobo *et al*., 2018) and in BD-II (Song *et al*., 2012; Holma *et al*., 2014) were reported, while other studies did not find significant differences between the two subtypes (Novick *et al*., 2010; Tondo *et al*., 2016). In the current meta-analysis, both BD-I and BD-NOS were positively associated while BD-II was negatively associated with lifetime prevalence of SA, which is similar to the findings of some (Angst *et al*., 2005; Popovic *et al*., 2015; Altamura *et al*., 2018), but not all (Angst *et al*., 2005; Neves *et al*., 2009; Pawlak *et al*., 2013) studies. The close association between the depressive phase and SA may be a reason for the diverse SA rates in different BD types (Schaffer *et al*., 2015*a*). SA is more likely to occur during mixed states and depressive episodes in BD-I, and during depressive episodes in BD-II (Tondo *et al*., 1999). Poorer insight and treatment adherence due to more frequent psychotic symptoms in BD-I (van der Werf-Eldering *et al*., 2011; Depp *et al*., 2014; Silva *et al*., 2015) can lead to an increased risk of SA.

In the subgroup analyses, the lifetime rate of SA in rapid cycling BD (47.0%) was significantly higher than in non-rapid cycling BD (34.4%). Rapid cycling is a major risk factor of SA in BD (MacKinnon *et al*., 2005), which is attributed to the younger age of onset, more illness severity, longer illness duration, poorer outcomes and higher risk of disability in rapid cycling BD (Kupka *et al*., 2005; Mackin, 2005; Schneck *et al*., 2008; Gigante *et al*., 2016). The lifetime rate of SA was significantly higher in high- and middle-income countries compared to low-income countries, and also higher in the Americas, Western Pacific and Europe than in Africa and South-East Asia. The discrepancy in SA rate across regions could be partly due to differences in health care services, ethnicity, and economic and sociocultural factors (Westman *et al*., 2003; Karch *et al*., 2006). Westman *et al*. (2003) have found that the place of birth and socioeconomic status were significantly associated with the risk of SA. The risk of suicide and associated factors were different between Caucasian and African Americans (Kung *et al*., 1998). Studies of suicide in BD in low- and middle-income countries are few and far between, which can result in biased comparisons.

The sample size was negatively correlated to the lifetime prevalence of SA. Results of studies with small sample size are less reliable (Dong *et al*., 2017). The higher 1-year prevalence of SA was found at baseline of cohort studies than in cross-sectional studies, while there was no group difference between lifetime and current episode prevalence between the two types of studies. Again, it is likely that the low number of cohort and cross-sectional studies resulted in unstable results.

The results of this meta-analysis should be interpreted with caution because of several methodological limitations. First, relevant factors related to SA, such as pharmacotherapy, comorbidities, illness severity and the actual mood state of BD, were not reported in most studies, hence their moderating effects on SA could not be explored. Second, publication bias of the 1-year and current episode prevalence of SA could not be assessed as the number of studies was <10 (Wan *et al*., 2013). In addition, the pooled current episode and 1-year SA prevalence estimates and the SA-moderating effects of the region and low-income countries were examined in a small number of studies, therefore the findings are only preliminary. Third, most studies were conducted in the Americas and Europe, making the generalisability of findings limited. Fourth, although subgroup analyses have been performed, heterogeneity could not be avoided as it is a common pitfall in the meta-analysis of epidemiological studies (Winsper *et al*., 2013; Long *et al*., 2014; Rotenstein *et al*., 2016). The heterogeneity in the current meta-analysis was probably due to the systematic differences between included studies, such as diverse study aims and inclusion/exclusion criteria. Fifth, there may be recall bias in the assessment of SA. Finally, the number of studies that reported data on the current episode and 1-year prevalence was less than those that examined lifetime prevalence that could have influenced the results to an uncertain degree.

In conclusion, the meta-analysis found that the prevalence of SA in BD is higher than the figures reported in schizophrenia and in the general population. Given the major impacts of SA on BD, more mental health resources should be allocated and effective measures should be undertaken to reduce the risk of SA in this population. Identifying the risk factors of SA (e.g. rapid cycling type and female gender as found in this study) and the long-term use of mood stabilisers coupled with psycho-social interventions could reduce the risk of suicidal behaviour (Rihmer, 2008), e.g. long-term treatment with lithium reduced SA by 10% and completed suicide by 20% (Benard *et al*., 2016).

## Supplementary material

For supplementary material accompanying this paper visit https://doi.org/10.1017/S2045796019000593. click here to view supplementary material

## Data

All the data supporting the findings of this meta-analysis have been provided in Tables, Figures and Supplemental Tables and Figures.

## References

[ref1] Altamura AC, Buoli M, Cesana B, Dell'Osso B, Tacchini G, Albert U, Fagiolini A, de Bartolomeis A, Maina G and Sacchetti E (2018) Socio-demographic and clinical characterization of patients with bipolar disorder I vs II: a Nationwide Italian Study. European Archives of Psychiatry and Clinical Neuroscience 268, 169–177. 28365865 10.1007/s00406-017-0791-0

[ref2] American Psychiatric Association (2013) Diagnostic and Statistical Manual of Mental Disorders (DSM-5®). Washington, DC: American Psychiatric Association.

[ref3] Angst J (2004) Bipolar disorder – a seriously underestimated health burden. European Archives of Psychiatry and Clinical Neuroscience 254, 59–60. 15146333 10.1007/s00406-004-0502-5

[ref4] Angst J, Angst F, Gerber-Werder R and Gamma A (2005) Suicide in 406 mood-disorder patients with and without long-term medication: a 40 to 44 years’ follow-up. Archives of Suicide Research 9, 279–300. 16020171 10.1080/13811110590929488

[ref5] Antypa N, Antonioli M and Serretti A (2013) Clinical, psychological and environmental predictors of prospective suicide events in patients with Bipolar Disorder. Journal of Psychiatric Research 47, 1800–1808. 24018102 10.1016/j.jpsychires.2013.08.005

[ref6] Baldessarini RJ, Pompili M and Tondo L (2006) Suicide in bipolar disorder: risks and management. CNS Spectrums 11, 465–471. 16816785 10.1017/s1092852900014681

[ref7] Baldessarini RJ, Innamorati M, Erbuto D, Serafini G, Fiorillo A, Amore M, Girardi P and Pompili M (2017) Differential associations of affective temperaments and diagnosis of major affective disorders with suicidal behavior. Journal of Affective Disorders 210, 19–21. 27992854 10.1016/j.jad.2016.12.003

[ref8] Bellivier F, Belzeaux R, Scott J, Courtet P, Golmard JL and Azorin JM (2017) Anticonvulsants and suicide attempts in bipolar I disorders. Acta Psychiatrica Scandinavica 135, 470–478. 28190254 10.1111/acps.12709

[ref9] Benard V, Vaiva G, Masson M and Geoffroy PA (2016) Lithium and suicide prevention in bipolar disorder. L'Encephale 42, 234–241. 10.1016/j.encep.2016.02.006 27000268

[ref10] Berk M, Dodd S, Callaly P, Berk L, Fitzgerald P, De Castella A, Filia S, Filia K, Tahtalian S and Biffin F (2007) History of illness prior to a diagnosis of bipolar disorder or schizoaffective disorder. Journal of Affective Disorders 103, 181–186. 17324469 10.1016/j.jad.2007.01.027

[ref11] Bernal M, Haro JM, Bernert S, Brugha T, de Graaf R, Bruffaerts R, Lepine JP, de Girolamo G, Vilagut G, Gasquet I, Torres JV, Kovess V, Heider D, Neeleman J, Kessler R and Alonso J (2007) Risk factors for suicidality in Europe: results from the ESEMED study. Journal of Affective Disorders 101, 27–34. 17074395 10.1016/j.jad.2006.09.018

[ref12] Beyer JL and Weisler RH (2016) Suicide behaviors in bipolar disorder: a review and update for the clinician. Psychiatric Clinics 39, 111–123. 26876322 10.1016/j.psc.2015.09.002

[ref13] Bezerra S, Galvao-de-Almeida A, Studart P, Martins DF, Caribe AC, Schwingel PA and Miranda-Scippa A (2017) Suicide attempts in bipolar I patients: impact of comorbid personality disorders. Revista Brasileira de Psiquiatria 39, 133–139. 28076649 10.1590/1516-4446-2016-1982 PMC7111445

[ref14] Bobo WV, Na PJ, Geske JR, McElroy SL, Frye MA and Biernacka JM (2018) The relative influence of individual risk factors for attempted suicide in patients with bipolar I versus bipolar II disorder. Journal of Affective Disorders 225, 489–494. 28865370 10.1016/j.jad.2017.08.076

[ref15] Boyle MH (1998) Guidelines for evaluating prevalence studies. Evidence-based Mental Health 1, 37–39.

[ref16] Brown GK, Beck AT, Steer RA and Grisham JR (2000) Risk factors for suicide in psychiatric outpatients: a 20-year prospective study. Journal of Consulting and Clinical Psychology 68, 371. 10883553

[ref17] Cao XL, Zhong BL, Xiang YT, Ungvari GS, Lai KY, Chiu HF and Caine ED (2015) Prevalence of suicidal ideation and suicide attempts in the general population of China: a meta-analysis. International Journal of Psychiatry in Medicine 49, 296–308. 26060259 10.1177/0091217415589306 PMC4536918

[ref18] Carballo JJ, Harkavy-Friedman J, Burke AK, Sher L, Baca-Garcia E, Sullivan GM, Grunebaum MF, Parsey RV, Mann JJ and Oquendo MA (2008) Family history of suicidal behavior and early traumatic experiences: additive effect on suicidality and course of bipolar illness? Journal of Affective Disorders 109, 57–63. 18221790 10.1016/j.jad.2007.12.225 PMC3491751

[ref19] Cremaschi L, Dell'Osso B, Vismara M, Dobrea C, Buoli M, Ketter TA and Altamura AC (2017) Onset polarity in bipolar disorder: a strong association between first depressive episode and suicide attempts. Journal of Affective Disorders 209, 182–187. 27936451 10.1016/j.jad.2016.11.043

[ref20] Cruz N, Vieta E, Comes M, Haro JM, Reed C and Bertsch J (2008) Rapid-cycling bipolar I disorder: course and treatment outcome of a large sample across Europe. Journal of Psychiatric Research 42, 1068–1075. 18262204 10.1016/j.jpsychires.2007.12.004

[ref21] Depp CA, Harmell AL, Savla GN, Mausbach BT, Jeste DV and Palmer BW (2014) A prospective study of the trajectories of clinical insight, affective symptoms, and cognitive ability in bipolar disorder. Journal of Affective Disorders 152, 250–255. 24200153 10.1016/j.jad.2013.09.020 PMC4011138

[ref22] Dong M, Wang SB, Wang F, Zhang L, Ungvari GS, Ng CH, Meng X, Yuan Z, Wang G and Xiang YT (2017) Suicide-related behaviours in schizophrenia in China: a comprehensive meta-analysis. Epidemiology and Psychiatric Sciences, 1–10. 10.1017/S2045796017000476 PMC6998905 28944747

[ref23] Dong M, Zeng LN, Lu L, Li XH, Ungvari GS, Ng CH, Chow IHI, Zhang L, Zhou Y and Xiang YT (2018) Prevalence of suicide attempt in individuals with major depressive disorder: a meta-analysis of observational surveys. Psychological Medicine, 1–14. 10.1017/S0033291718002301 30178722

[ref24] Drake RE, Gates C, Whitaker A and Cotton PG (1985) Suicide among schizophrenics: a review. Comprehensive Psychiatry 26, 90–100. 3881217 10.1016/0010-440x(85)90053-7

[ref25] Duko B and Ayano G (2018) Suicidal ideation and attempts among people with severe mental disorder, Addis Ababa, Ethiopia, comparative cross-sectional study. Annals of General Psychiatry 17, 23. 29881440 10.1186/s12991-018-0193-3 PMC5984440

[ref26] Elizabeth Sublette M, Carballo JJ, Moreno C, Galfalvy HC, Brent DA, Birmaher B, John Mann J and Oquendo MA (2009) Substance use disorders and suicide attempts in bipolar subtypes. Journal of Psychiatric Research 43, 230–238. 18590916 10.1016/j.jpsychires.2008.05.001 PMC2671238

[ref27] Gigante AD, Barenboim IY, Dias RD, Toniolo RA, Mendonca T, Miranda-Scippa A, Kapczinski F and Lafer B (2016) Psychiatric and clinical correlates of rapid cycling bipolar disorder: a cross-sectional study. Revista Brasileira de Psiquiatria 38, 270–274. 27304255 10.1590/1516-4446-2015-1789 PMC7111346

[ref28] Gonda X, Pompili M, Serafini G, Montebovi F, Campi S, Dome P, Duleba T, Girardi P and Rihmer Z (2012) Suicidal behavior in bipolar disorder: epidemiology, characteristics and major risk factors. Journal of Affective Disorders 143, 16–26. 22763038 10.1016/j.jad.2012.04.041

[ref29] Goodwin FK and Jamison KR (2007) Manic-depressive Illness: Bipolar Disorders and Recurrent Depression. United Kingdom: Oxford University Press.

[ref30] Harkavy-Friedman JM, Restifo K, Malaspina D, Kaufmann CA, Amador XF, Yale SA and Gorman JM (1999) Suicidal behavior in schizophrenia: characteristics of individuals who had and had not attempted suicide. American Journal of Psychiatry 156, 1276–1278. 10.1176/ajp.156.8.1276 10450275

[ref31] Hawton K, Sutton L, Haw C, Sinclair J and Harriss L (2005) Suicide and attempted suicide in bipolar disorder: a systematic review of risk factors. The Journal of Clinical Psychiatry 66, 693–704. 15960561 10.4088/jcp.v66n0604

[ref32] Higgins JP and Green S (2008) Cochrane handbook for systematic reviews of interventions. The Cochrane Collaboration.

[ref33] Holma KM, Haukka J, Suominen K, Valtonen HM, Mantere O, Melartin TK, Sokero TP, Oquendo MA and Isometsa ET (2014) Differences in incidence of suicide attempts between bipolar I and II disorders and major depressive disorder. Bipolar Disorders 16, 652–661. 24636453 10.1111/bdi.12195

[ref34] Howe AS, Leung T, Bani-Fatemi A, Souza R, Tampakeras M, Zai C, Kennedy JL, Strauss J and De Luca V (2014) Lack of association between dopamine-beta hydroxylase gene and a history of suicide attempt in schizophrenia: comparison of molecular and statistical haplotype analyses. Psychiatric Genetics 24, 110–115. 24710129 10.1097/YPG.0000000000000031

[ref35] Karch DL, Barker L and Strine TW (2006) Race/ethnicity, substance abuse, and mental illness among suicide victims in 13 US states: 2004 data from the National Violent Death Reporting System. Injury Prevention 12(Suppl 2), ii22–ii27. 17170166 10.1136/ip.2006.013557 PMC2563485

[ref36] Kattimani S, Subramanian K, Sarkar S, Rajkumar RP and Balasubramanian S (2017) Lifetime suicide attempt in bipolar I disorder: its correlates and effect on illness course. International Journal of Psychiatry in Clinical Practice 21, 118–124. 27854557 10.1080/13651501.2016.1250912

[ref37] Kessler RC, Berglund P, Borges G, Nock M and Wang PS (2005) Trends in suicide ideation, plans, gestures, and attempts in the United States, 1990–1992 to 2001–2003. JAMA 293, 2487–2495. 15914749 10.1001/jama.293.20.2487

[ref38] Kung KC, Liu X and Juon HS (1998) Risk factors for suicide in Caucasians and in African-Americans: a matched case-control study. Social Psychiatry and Psychiatric Epidemiology 33, 155–161. 9567665 10.1007/s001270050038

[ref39] Kupka RW, Luckenbaugh DA, Post RM, Suppes T, Altshuler LL, Keck PE Jr, Frye MA, Denicoff KD, Grunze H, Leverich GS, McElroy SL, Walden J and Nolen WA (2005) Comparison of rapid-cycling and non-rapid-cycling bipolar disorder based on prospective mood ratings in 539 outpatients. American Journal of Psychiatry 162, 1273–1280. 10.1176/appi.ajp.162.7.1273 15994709

[ref40] Latalova K, Kamaradova D and Prasko J (2014) Suicide in bipolar disorder: a review. Psychiatria Danubina 26, 108–114. 24909246

[ref41] Leverich GS, Altshuler LL, Frye MA, Suppes T, Keck PE, McElroy SL, Denicoff KD, Obrocea G, Nolen WA, Kupka R, Walden J, Grunze H, Perez S, Luckenbaugh DA and Post RM (2003) Factors associated with suicide attempts in 648 patients with bipolar disorder in the Stanley Foundation Bipolar Network. Journal of Clinical Psychiatry 64, 506–515. 10.4088/jcp.v64n0503 12755652

[ref42] Loney PL, Chambers LW, Bennett KJ, Roberts JG and Stratford PW (1998) Critical appraisal of the health research literature: prevalence or incidence of a health problem. Chronic Diseases in Canada 19, 170–176. 10029513

[ref43] Long J, Huang G, Liang W, Liang B, Chen Q, Xie J, Jiang J and Su L (2014) The prevalence of schizophrenia in mainland China: evidence from epidemiological surveys. Acta Psychiatrica Scandinavica 130, 244–256. 24916190 10.1111/acps.12296

[ref44] Mackin P (2005) Rapid cycling is equivalently prevalent in bipolar I and bipolar II disorder, and is associated with female gender and greater severity of illness. Evidence-based Mental Health 8, 52. 15851815 10.1136/ebmh.8.2.52

[ref45] MacKinnon DF, Potash JB, McMahon FJ, Simpson SG, Raymond Depaulo J Jr, Initiative NIoMHBDG and Zandi PP (2005) Rapid mood switching and suicidality in familial bipolar disorder. Bipolar Disorders 7, 441–448. 16176437 10.1111/j.1399-5618.2005.00236.x

[ref46] Matte DL, Pizzichini MM, Hoepers AT, Diaz AP, Karloh M, Dias M and Pizzichini E (2016) Prevalence of depression in COPD: a systematic review and meta-analysis of controlled studies. Respiratory Medicine 117, 154–161. 27492526 10.1016/j.rmed.2016.06.006

[ref47] Merikangas KR, Jin R, He J-P, Kessler RC, Lee S, Sampson NA, Viana MC, Andrade LH, Hu C and Karam EG (2011) Prevalence and correlates of bipolar spectrum disorder in the world mental health survey initiative. Archives of General Psychiatry 68, 241–251. 21383262 10.1001/archgenpsychiatry.2011.12 PMC3486639

[ref48] Neves FS, Malloy-Diniz LF and Correa H (2009) Suicidal behavior in bipolar disorder: what is the influence of psychiatric comorbidities? Journal of Clinical Psychiatry 70, 13–18. 10.4088/jcp.08m04037 19026263

[ref49] Nierenberg AA, Gray SM and Grandin LD (2001) Mood disorders and suicide. The Journal of Clinical Psychiatry. 11765092

[ref50] Novick DM, Swartz HA and Frank E (2010) Suicide attempts in bipolar I and bipolar II disorder: a review and meta-analysis of the evidence. Bipolar Disorders 12, 1–9. 10.1111/j.1399-5618.2009.00786.x PMC4536929 20148862

[ref51] Oquendo MA, Waternaux C, Brodsky B, Parsons B, Haas GL, Malone KM and Mann JJ (2000) Suicidal behavior in bipolar mood disorder: clinical characteristics of attempters and nonattempters. Journal of Affective Disorders 59, 107–117. 10837879 10.1016/s0165-0327(99)00129-9

[ref52] Oquendo MA, Currier D, Liu SM, Hasin DS, Grant BF and Blanco C (2010) Increased risk for suicidal behavior in comorbid bipolar disorder and alcohol use disorders: results from the National Epidemiologic Survey on Alcohol and Related Conditions (NESARC). Journal of Clinical Psychiatry 71, 902–909. 10.4088/JCP.09m05198gry PMC2914308 20667292

[ref53] Passos IC, Jansen K, Cardoso TD, Colpo GD, Zeni CP, Quevedo J, Kauer-Sant'Anna M, Zunta-Soares G, Soares JC and Kapczinski F (2016) Clinical outcomes associated With comorbid posttraumatic stress disorder among patients with bipolar disorder. Journal of Clinical Psychiatry 77, E555–E560. 10.4088/JCP.15m09935 27135375

[ref54] Patel R, Shetty H, Jackson R, Broadbent M, Stewart R, Boydell J, McGuire P and Taylor M (2015) Delays before diagnosis and initiation of treatment in patients presenting to mental health services with bipolar disorder. PLoS ONE 10, e0126530. 25992560 10.1371/journal.pone.0126530 PMC4439113

[ref55] Pawlak J, Dmitrzak-Weglarz M, Skibinska M, Szczepankiewicz A, Leszczynska-Rodziewicz A, Rajewska-Rager A, Maciukiewicz M, Czerski P and Hauser J (2013) Suicide attempts and psychological risk factors in patients with bipolar and unipolar affective disorder. General Hospital Psychiatry 35, 309–313. 23352318 10.1016/j.genhosppsych.2012.11.010

[ref56] Perlis RH (2005) Misdiagnosis of bipolar disorder. The American Journal of Managed Care 11, S271–S274. 16232009

[ref57] Plans L, Barrot C, Nieto E, Rios J, Schulze TG, Papiol S, Mitjans M, Vieta E and Benabarre A (2019) Association between completed suicide and bipolar disorder: a systematic review of the literature. Journal of Affective Disorders 242, 111–122. 30173059 10.1016/j.jad.2018.08.054

[ref58] Pompili M, Rihmer Z, Innamorati M, Lester D, Girardi P and Tatarelli R (2009) Assessment and treatment of suicide risk in bipolar disorders. Expert Review of Neurotherapeutics 9, 109–136. 19102673 10.1586/14737175.9.1.109

[ref59] Popovic D, Vieta E, Azorin JM, Angst J, Bowden CL, Mosolov S, Young AH and Perugi G (2015) Suicide attempts in major depressive episode: evidence from the BRIDGE-II-Mix study. Bipolar Disorders 17, 795–803. 26415692 10.1111/bdi.12338

[ref60] Rihmer Z (2008) Prediction and prevention of suicide in mood disorders. European Psychiatry 23, S45.

[ref61] Rosa AR, Cruz N, Franco C, Haro JM, Bertsch J, Reed C, Aarre TF, Sanchez-Moreno J and Vieta E (2010) Why do clinicians maintain antidepressants in some patients with acute mania? Hints from the European Mania in Bipolar Longitudinal Evaluation of Medication (EMBLEM), a large naturalistic study. The Journal of Clinical Psychiatry 71, 1000–1006. 20361912 10.4088/JCP.09m05026gre

[ref62] Rotenstein LS, Ramos MA, Torre M, Segal JB, Peluso MJ, Guille C, Sen S and Mata DA (2016) Prevalence of depression, depressive symptoms, and suicidal ideation among medical students: a systematic review and meta-analysis. JAMA 316, 2214–2236. 27923088 10.1001/jama.2016.17324 PMC5613659

[ref63] Sachs GS, Thase ME, Otto MW, Bauer M, Miklowitz D, Wisniewski SR, Lavori P, Lebowitz B, Rudorfer M and Frank E (2003) Rationale, design, and methods of the systematic treatment enhancement program for bipolar disorder (STEP-BD). Biological Psychiatry 53, 1028–1042. 12788248 10.1016/s0006-3223(03)00165-3

[ref64] Schaffer A, Isometsä ET, Azorin J-M, Cassidy F, Goldstein T, Rihmer Z, Sinyor M, Tondo L, Moreno DH and Turecki G (2015 *a*) A review of factors associated with greater likelihood of suicide attempts and suicide deaths in bipolar disorder: part II of a report of the International Society for Bipolar Disorders Task Force on Suicide in Bipolar Disorder. Australian and New Zealand Journal of Psychiatry 49, 1006–1020. 10.1177/0004867415594428 PMC5858693 26175498

[ref65] Schaffer A, Isometsä ET, Tondo L, Moreno DH, Turecki G, Reis C, Cassidy F, Sinyor M, Azorin JM and Kessing LV (2015 *b*) International Society for Bipolar Disorders Task Force on Suicide: meta-analyses and meta-regression of correlates of suicide attempts and suicide deaths in bipolar disorder. Bipolar Disorders 17, 1–16. 10.1111/bdi.12271 PMC6296224 25329791

[ref66] Schneck CD, Miklowitz DJ, Calabrese JR, Allen MH, Thomas MR, Wisniewski SR, Miyahara S, Shelton MD, Ketter TA and Goldberg JF (2004) Phenomenology of rapid-cycling bipolar disorder: data from the first 500 participants in the Systematic Treatment Enhancement Program. American Journal of Psychiatry 161, 1902–1908. 10.1176/ajp.161.10.1902 15465989

[ref67] Schneck CD, Miklowitz DJ, Miyahara S, Araga M, Wisniewski S, Gyulai L, Allen MH, Thase ME and Sachs GS (2008) The prospective course of rapid-cycling bipolar disorder: findings from the STEP-BD. American Journal of Psychiatry 165, 370–377, quiz 410. 10.1176/appi.ajp.2007.05081484 18198271

[ref68] Shabani A, Teimurinejad S, Kokar S, Ahmadzad Asl M, Shariati B, Mousavi Behbahani Z, Ghasemzadeh MR, Hasani S, Taban M, Shirekhoda S, Ghorbani Z, Tat S, Nohesara S and Shariat SV (2013) Suicide risk factors in Iranian patients with bipolar disorder: a 21-month follow-up from BDPF study. Iranian Journal of Psychiatry and Behavioral Sciences 7, 16–23. PMC3939979 24644495

[ref69] Silva RdAd, Mograbi DC, Camelo EV, Bifano J, Wainstok M, Silveira LAS and Cheniaux E (2015) Insight in bipolar disorder: a comparison between mania, depression and euthymia using the Insight Scale for Affective Disorders. Trends in Psychiatry and Psychotherapy 37, 152–156. 26630406 10.1590/2237-6089-2015-0014

[ref70] Simon GE, Hunkeler E, Fireman B, Lee JY and Savarino J (2007) Risk of suicide attempt and suicide death in patients treated for bipolar disorder. Bipolar Disorders 9, 526–530. 17680924 10.1111/j.1399-5618.2007.00408.x

[ref71] Song JY, Yu HY, Kim SH, Hwang SSH, Cho HS, Kim YS, Ha K and Ahn YM (2012) Assessment of risk factors related to suicide attempts in patients with bipolar disorder. Journal of Nervous and Mental Disease 200, 978–984. 10.1097/NMD.0b013e3182718a07 23124183

[ref72] Spitzer RL, Endicott J and Robins E (1978) Research diagnostic criteria: rationale and reliability. Archives of General Psychiatry 35, 773–782. 655775 10.1001/archpsyc.1978.01770300115013

[ref73] Stefanello S, Cais CFdS, Mauro MLF, Freitas GVSd and Botega NJ (2008) Gender differences in suicide attempts: preliminary results of the multisite intervention study on suicidal behavior (SUPRE-MISS) from Campinas, Brazil. Revista Brasileira de Psiquiatria 30, 139–143. 18176725 10.1590/s1516-44462006005000063

[ref74] Stroup DF, Berlin JA, Morton SC, Olkin I, Williamson GD, Rennie D, Moher D, Becker BJ, Sipe TA and Thacker SB (2000) Meta-analysis of observational studies in epidemiology: a proposal for reporting. Meta-analysis Of Observational Studies in Epidemiology (MOOSE) group. JAMA 283, 2008–2012. 10789670 10.1001/jama.283.15.2008

[ref75] Studart P, Galvao-de Almeida A, Bezerra S, Caribe A, Afonso NR, Daltro C and Miranda-Scippa AA (2016) Is history of suicidal behavior related to social support and quality of life in outpatients with bipolar I disorder? Psychiatry Research 246, 796–802. 28029441 10.1016/j.psychres.2016.10.045

[ref76] Swann AC, Dougherty DM, Pazzaglia PJ, Pham M, Steinberg JL and Moeller FG (2005) Increased impulsivity associated with severity of suicide attempt history in patients with bipolar disorder. American Journal of Psychiatry 162, 1680–1687. 10.1176/appi.ajp.162.9.1680 16135628

[ref77] Szadoczky E, Vitrai J, Rihmer Z and Furedi J (2000) Suicide attempts in the Hungarian adult population. Their relation with DIS/DSM-III-R affective and anxiety disorders. European Psychiatry 15, 343–347. 11004728 10.1016/s0924-9338(00)90501-7

[ref78] Tondo L and Baldessarini RJ (2005) Suicidal risk in bipolar disorder. Clinical Neuropsychiatry 2, 55–65.

[ref79] Tondo L, Baldessarini RJ, Hennen J, Minnai GP, Salis P, Scamonatti L, Masia M, Ghiani C and Mannu P (1999) Suicide attempts in major affective disorder patients with comorbid substance use disorders. Journal of Clinical Psychiatry 60(Suppl 2), 63–69; discussion 75-66, 113–116. 10073390

[ref80] Tondo L, Isacsson G and Baldessarini R (2003) Suicidal behaviour in bipolar disorder: risk and prevention. CNS Drugs 17, 491–511. 12751919 10.2165/00023210-200317070-00003

[ref81] Tondo L, Pompili M, Forte A and Baldessarini RJ (2016) Suicide attempts in bipolar disorders: comprehensive review of 101 reports. Acta Psychiatrica Scandinavica 133, 174–186. 26555604 10.1111/acps.12517

[ref82] van der Werf-Eldering MJ, van der Meer L, Burger H, Holthausen EA, Nolen WA and Aleman A (2011) Insight in bipolar disorder: associations with cognitive and emotional processing and illness characteristics. Bipolar Disorders 13, 343–354. 21843274 10.1111/j.1399-5618.2011.00934.x

[ref83] Walker J, Holm Hansen C, Martin P, Sawhney A, Thekkumpurath P, Beale C, Symeonides S, Wall L, Murray G and Sharpe M (2013) Prevalence of depression in adults with cancer: a systematic review. Annals of Oncology 24, 895–900. 23175625 10.1093/annonc/mds575

[ref84] Wan Y, Hu Q, Li T, Jiang L, Du Y, Feng L, Wong JC-M and Li C (2013) Prevalence of autism spectrum disorders among children in China: a systematic review. Shanghai Archives of Psychiatry 25, 70. 24991138 10.3969/j.issn.1002-0829.2013.02.003 PMC4054540

[ref85] Westman J, Hasselstrom J, Johansson SE and Sundquist J (2003) The influences of place of birth and socioeconomic factors on attempted suicide in a defined population of 4.5 million people. Archives of General Psychiatry 60, 409–414. 12695319 10.1001/archpsyc.60.4.409

[ref86] Winsper C, Ganapathy R, Marwaha S, Large M, Birchwood M and Singh S (2013) A systematic review and meta-regression analysis of aggression during the First Episode of Psychosis. Acta Psychiatrica Scandinavica 128, 413–421. 23521361 10.1111/acps.12113

[ref87] Yang C, Zhang L, Zhu P, Zhu C and Guo Q (2016) The prevalence of tic disorders for children in China: a systematic review and meta-analysis. Medicine (Baltimore) 95, e4354. 27472724 10.1097/MD.0000000000004354 PMC5265861

