# Supplementary material for: Prevalence of suicide attempts in bipolar disorder: a systematic review and meta-analysis of observational studies
Source: Epidemiol Psychiatr Sci. 2019 Oct 25;29:e63. doi: 10.1017/S2045796019000593 (PMC8061290; doi:10.1017/S2045796019000593)
Supplement: Supplementary file 1 [file epssup.zip › S2045796019000593sup002.docx]

**References**

**Aas, M., Etain, B., Bellivier, F., Henry, C., Lagerberg, T., Ringen, A., Agartz, I., Gard, S., Kahn, J. P., Leboyer, M., Andreassen, O. A. & Melle, I.** (2014). Additive effects of childhood abuse and cannabis abuse on clinical expressions of bipolar disorders. *Psychological Medicine* **44**, 1653-1662.

**Akiskal, H. S., Hantouche, E. G., Allilaire, J. F., Sechter, D., Bourgeois, M. L., Azorin, J. M., Chatenet-Duchene, L. & Lancrenon, S.** (2003). Validating antidepressant-associated hypomania (bipolar III): a systematic comparison with spontaneous hypomania (bipolar II). *Journal of Affective Disorders* **73**, 65-74.

**Altamura, A. C., Buoli, M., Cesana, B., Dell'Osso, B., Tacchini, G., Albert, U., Fagiolini, A., de Bartolomeis, A., Maina, G. & Sacchetti, E.** (2018). Socio-demographic and clinical characterization of patients with Bipolar Disorder I vs II: a nationwide Italian study. *European Archives of Psychiatry and Clinical Neuroscience* **268**, 169-177.

**Azorin, J. M., Kaladjian, A., Adida, M., Hantouche, E., Hameg, A., Lancrenon, S. & Akiskal, H. S.** (2008). Toward the delineation of mania subtypes in the French National EPIMAN-II Mille Cohort. *European Archives of Psychiatry and Clinical Neuroscience* **258**, 497-504.

**Baldessarini, R. J., Innamorati, M., Erbuto, D., Serafini, G., Fiorillo, A., Amore, M., Girardi, P. & Pompili, M.** (2017). Differential associations of affective temperaments and diagnosis of major affective disorders with suicidal behavior. *Journal of Affective Disorders* **210**, 19-21.

**Baldessarini, R. J., Tondo, L., Vazquez, G. H., Undurraga, J., Bolzani, L., Yildiz, A., Khalsa, H. M., Lai, M., Lepri, B., Lolich, M., Maffei, P. M., Salvatore, P., Faedda, G. L., Vieta, E. & Tohen, M.** (2012). Age at onset versus family history and clinical outcomes in 1,665 international bipolar-I disorder patients. *World Psychiatry* **11**, 40-6.

**Bani-Fatemi, A., Polsinelli, G., Kennedy, J. L. & De Luca, V.** (2013). Ethnicity and suicide attempt: analysis in bipolar disorder and schizophrenia. *BMC Psychiatry* **13**, 252.

**Bellivier, F., Belzeaux, R., Scott, J., Courtet, P., Golmard, J. L. & Azorin, J. M.** (2017). Anticonvulsants and suicide attempts in bipolar I disorders. *Acta Psychiatrica Scandinavica* **135**, 470-478.

**Bellivier, F., Leboyer, M., Courtet, P., Buresi, C., Beaufils, B., Samolyk, D., Allilaire, J. F., Feingold, J., Mallet, J. & Malafosse, A.** (1998). Association between the tryptophan hydroxylase gene and manic-depressive illness. *Archives of General Psychiatry* **55**, 33-7.

**Benedetti, F., Riccaboni, R., Poletti, S., Radaelli, D., Locatelli, C., Lorenzi, C., Pirovano, A., Smeraldi, E. & Colombo, C.** (2014). The serotonin transporter genotype modulates the relationship between early stress and adult suicidality in bipolar disorder. *Bipolar Disorders* **16**, 857-66.

**Berkol, T. D., Islam, S., Kirli, E., Pinarbasi, R. & Ozyildirim, I.** (2016). Suicide attempts and clinical features of bipolar patients. *Saudi Medical Journal* **37**, 662-7.

**Berutti, M., Nery, F. G., Sato, R., Scippa, A., Kapczinski, F. & Lafer, B.** (2014). Association between family history of mood disorders and clinical characteristics of bipolar disorder: results from the Brazilian bipolar research network. *Journal of Affective Disorders* **161**, 104-8.

**Bezerra, S., Galvao-de-Almeida, A., Studart, P., Martins, D. F., Caribe, A. C., Schwingel, P. A. & Miranda-Scippa, A.** (2017). Suicide attempts in bipolar I patients: impact of comorbid personality disorders. *Revista Brasileira de Psiquiatria* **39**, 133-139.

**Bobo, W. V., Na, P. J., Geske, J. R., McElroy, S. L., Frye, M. A. & Biernacka, J. M.** (2018). The relative influence of individual risk factors for attempted suicide in patients with bipolar I versus bipolar II disorder. *Journal of Affective Disorders* **225**, 489-494.

**Bolton, J. M. & Robinson, J.** (2010). Population-attributable fractions of Axis I and Axis II mental disorders for suicide attempts: Findings from a representative sample of the adult, noninstitutionalized US population. *American Journal of Public Health* **100**, 2473-2480.

**Brietzke, E., Moreira, C., Duarte, S. V. B., Nery, F. G., Kapczinski, F., Scippa, A. M. & Lafer, B.** (2012). Impact of comorbid migraine on the clinical course of bipolar disorder. *Comprehensive Psychiatry* **53**, 809-812.

**Cardoso, B. M., Sant' Anna, M. K., Dias, V. V., Andreazza, A. C., Ceresér, K. M. & Kapczinski, F.** (2008). The impact of co-morbid alcohol use disorder in bipolar patients. *Alcohol* **42**, 451-457.

**Caribe, A. C., Studart, P., Bezerra, S., Brietzke, E., Noto, M. N., Vianna-Sulzbach, M., Kapczinski, F., Neves, F. S., Correa, H. & Miranda-Scippa, A.** (2015). Is religiosity a protective factor against suicidal behavior in bipolar I outpatients? *Journal of Affective Disorders* **186**, 156-161.

**Chen, Y. W. & Dilsaver, S. C.** (1996). Lifetime rates of suicide attempts among subjects with bipolar and unipolar disorders relative to subjects with other Axis I disorders. *Biological Psychiatry* **39**, 896-9.

**Comtois, K. A., Russo, J. E., Roy-Byrne, P. & Ries, R. K.** (2004). Clinicians' Assessments of Bipolar Disorder and Substance Abuse as Predictors of Suicidal Behavior in Acutely Hospitalized Psychiatric Inpatients. *Biological Psychiatry* **56**, 757-763.

**Coryell, W., Fiedorowicz, J., Leon, A. C., Endicott, J. & Keller, M. B.** (2013). Age of onset and the prospectively observed course of illness in bipolar disorder. *Journal of Affective Disorders* **146**, 34-38.

**Cremaschi, L., Dell'Osso, B., Vismara, M., Dobrea, C., Buoli, M., Ketter, T. A. & Altamura, A. C.** (2017). Onset polarity in bipolar disorder: A strong association between first depressive episode and suicide attempts. *Journal of Affective Disorders* **209**, 182-187.

**D'Ambrosio, V., Salvi, V., Bogetto, F. & Maina, G.** (2012). Serum lipids, metabolic syndrome and lifetime suicide attempts in patients with bipolar disorder. *Progress in Neuro-Psychopharmacology and Biological Psychiatry* **37**, 136-40.

**Dalton, E. J., Cate-Carter, T. D., Mundo, E., Parikh, S. V. & Kennedy, J. L.** (2003). Suicide risk in bipolar patients: the role of co-morbid substance use disorders. *Bipolar Disorders* **5**, 58-61.

**de Abreu, L. N., Nery, F. G., Harkavy-Friedman, J. M., de Almeida, K. M., Gomes, B. C., Oquendo, M. A. & Lafer, B.** (2012). Suicide attempts are associated with worse quality of life in patients with bipolar disorder type I. *Comprehensive Psychiatry* **53**, 125-9.

**Dell'Osso, B., Holtzman, J. N., Goffin, K. C., Portillo, N., Hooshmand, F., Miller, S., Dore, J., Wang, P. W., Hill, S. J. & Ketter, T. A.** (2015). American tertiary clinic-referred bipolar II disorder compared to bipolar I disorder: More severe in multiple ways, but less severe in a few other ways. *Journal of Affective Disorders* **188**, 257-62.

**Dervic, K., Carballo, J. J., Baca-Garcia, E., Galfalvy, H. C., Mann, J. J., Brent, D. A. & Oquendo, M. A.** (2011). Moral or Religious Objections to Suicide May Protect Against Suicidal Behavior in Bipolar Disorder. *Journal of Clinical Psychiatry* **72**, 1390-1396.

**Duko, B. & Ayano, G.** (2018). Suicidal ideation and attempts among people with severe mental disorder, Addis Ababa, Ethiopia, comparative cross-sectional study. *Annals of General Psychiatry* **17**, 23.

**Endicott, J., Nee, J., Andreasen, N., Clayton, P., Keller, M. & Coryell, W.** (1985). Bipolar II. Combine or keep separate? *Journal of Affective Disorders* **8**, 17-28.

**Engström, C., Brändström, S., Sigvardsson, S., Cloninger, C. R. & Nylander, P. O.** (2004). Bipolar disorder. III: Harm avoidance a risk factor for suicide attempts. *Bipolar Disorders* **6**, 130-138.

**Eroglu, M. Z., Karakus, G. & Tamam, L.** (2013). Bipolar disorder and suicide. *Dusunen Adam* **26**, 139-147.

**Fiedorowicz, J. G., Leon, A. C., Keller, M. B., Solomon, D. A., Rice, J. P. & Coryell, W. H.** (2009). Do risk factors for suicidal behavior differ by affective disorder polarity? *Psychological Medicine* **39**, 763-771.

**Finseth, P. I., Sonderby, I. E., Djurovic, S., Agartz, I., Malt, U. F., Melle, I., Morken, G., Andreassen, O. A., Vaaler, A. E. & Tesli, M.** (2014). Association analysis between suicidal behaviour and candidate genes of bipolar disorder and schizophrenia. *Journal of Affective Disorders* **163**, 110-4.

**Furlong, R. A., Ho, L., Rubinsztein, J. S., Walsh, C., Paykel, E. S. & Rubinsztein, D. C.** (1998). No association of the tryptophan hydroxylase gene with bipolar affective disorder, unipolar affective disorder, or suicidal behaviour in major affective disorder. *American Journal of Medical Genetics* **81**, 245-7.

**Ghanizadeh, A. & Sahraian, A.** (2008). Suicidal attempters and non-attempters with bipolar disorder in Iran. *Depression and Anxiety* **25**, E111-4.

**Gigante, A. D., Barenboim, I. Y., Dias, R. D., Toniolo, R. A., Mendonca, T., Miranda-Scippa, A., Kapczinski, F. & Lafer, B.** (2016). Psychiatric and clinical correlates of rapid cycling bipolar disorder: a cross-sectional study. *Revista Brasileira de Psiquiatria* **38**, 270-274.

**Gomes, F. A., Kauer-Sant'Anna, M., Magalhaes, P. V., Jacka, F. N., Dodd, S., Gama, C. S., Cunha, A., Berk, M. & Kapczinski, F.** (2010). Obesity is associated with previous suicide attempts in bipolar disorder. *Acta Neuropsychiatrica* **22**, 63-67.

**Gonzalez, V. M.** (2008). Recognition of mental illness and suicidality among individuals with serious mental illness. *Journal of Nervous and Mental Disease* **196**, 727-34.

**Henry, C., Etain, B., Godin, O., Dargel, A. A., Azorin, J. M., Gard, S., Bellivier, F., Bougerol, T., Kahn, J. P., Passerieux, C., Aubin, V., Courtet, P. & Leboyer, M.** (2015). Bipolar patients referred to specialized services of care: Not resistant but impaired by sub-syndromal symptoms. Results from the FACE-BD cohort. *Australian and New Zealand Journal of Psychiatry* **49**, 898-905.

**Henry, C., Van den Bulke, D., Bellivier, F., Etain, B., Rouillon, F. & Leboyer, M.** (2003). Anxiety disorders in 318 bipolar patients: Prevalence and impact on illness severity and response to mood stabilizer. *Journal of Clinical Psychiatry* **64**, 331-335.

**Holma, K. M., Haukka, J., Suominen, K., Valtonen, H. M., Mantere, O., Melartin, T. K., Sokero, T. P., Oquendo, M. A. & Isometsa, E. T.** (2014). Differences in incidence of suicide attempts between bipolar I and II disorders and major depressive disorder. *Bipolar Disorders* **16**, 652-661.

**Izci, F., Findikli, E. K., Zincir, S., Zincir, S. B. & Koc, M. I.** (2016). The differences in temperament-character traits, suicide attempts, impulsivity, and functionality levels of patients with bipolar disorder I and II. *Neuropsychiatric Disease and Treatment* **12**, 177-84.

**Jimenez, E., Arias, B., Mitjans, M., Goikolea, J. M., Ruiz, V., Brat, M., Saiz, P. A., Garcia-Portilla, M. P., Buron, P., Bobes, J., Oquendo, M. A., Vieta, E. & Benabarre, A.** (2016). Clinical features, impulsivity, temperament and functioning and their role in suicidality in patients with bipolar disorder. *Acta Psychiatrica Scandinavica* **133**, 266-276.

**Joyce, P. R., Light, K. J., Rowe, S. L., Cloninger, C. R. & Kennedy, M. A.** (2010). Self-mutilation and suicide attempts: Relationships to bipolar disorder, borderline personality disorder, temperament and character. *Australian and New Zealand Journal of Psychiatry* **44**, 250-257.

**Karakus, G. & Tamam, L.** (2011). Impulse control disorder comorbidity among patients with bipolar I disorder. *Comprehensive Psychiatry* **52**, 378-85.

**Kattimani, S., Subramanian, K., Sarkar, S., Rajkumar, R. P. & Balasubramanian, S.** (2017). Lifetime suicide attempt in bipolar I disorder: Its correlates and effect on illness course. *International Journal of Psychiatry in Clinical Practice* **21**, 118-124.

**Kenneson, A., Funderburk, J. S. & Maisto, S. A.** (2013). Risk factors for secondary substance use disorders in people with childhood and adolescent-onset bipolar disorder: opportunities for prevention. *Comprehensive Psychiatry* **54**, 439-46.

**Kim, B., Kim, C. Y., Hong, J. P., Kim, S. Y., Lee, C. & Joo, Y. H.** (2008). Brain-Derived Neurotrophic Factor Val/Met Polymorphism and Bipolar Disorder - Association of the Met Allele with Suicidal Behavior of Bipolar Patients. *Neuropsychobiology* **58**, 97-103.

**Kim, J. S., Ha, T. H., Chang, J. S., Park, Y. S., Huh, I., Kim, J., Hong, K. S., Park, T. & Ha, K.** (2015). Seasonality and its distinct clinical correlates in bipolar II disorder. *Psychiatry Research* **225**, 540-4.

**Kruger, S., Braunig, P. & Cooke, R. G.** (2000). Comorbidity of obsessive-compulsive disorder in recovered inpatients with bipolar disorder. *Bipolar Disorders* **2**, 71-74.

**Kvitland, L. R., Melle, I., Aminoff, S. R., Lagerberg, T. V., Andreassen, O. A. & Ringen, P. A.** (2016). Cannabis use in first-treatment bipolar I disorder: relations to clinical characteristics. *Early Intervention in Psychiatry* **10**, 36-44.

**Leverich, G. S., McElroy, S. L., Suppes, T., Keck Jr, P. E., Denicoff, K. D., Nolen, W. A., Altshuler, L. L., Rush, A. J., Kupka, R., Frye, M. A., Autio, K. A. & Post, R. M.** (2002). Early physical and sexual abuse associated with an adverse course of bipolar illness. *Biological Psychiatry* **51**, 288-297.

**Lopez, P., Mosquera, F., de Leon, J., Gutierrez, M., Ezcurra, J., Ramirez, F. & Gonzalez-Pinto, A.** (2001). Suicide attempts in bipolar patients. *Journal of Clinical Psychiatry* **62**, 963-6.

**Manchia, M., Lampus, S., Chillotti, C., Sardu, C., Ardau, R., Severino, G. & Del Zompo, M.** (2008). Age at onset in Sardinian bipolar I patients: Evidence for three subgroups. *Bipolar Disorders* **10**, 443-446.

**Mitchell, P. B., Frankland, A., Hadzi-Pavlovic, D., Roberts, G., Corry, J., Wright, A., Loo, C. K. & Breakspear, M.** (2011). Comparison of depressive episodes in bipolar disorder and in major depressive disorder within bipolar disorder pedigrees. *British Journal of Psychiatry* **199**, 303-9.

**Mitchell, P. B., Johnston, A. K., Corry, J., Ball, J. R. & Malhi, G. S.** (2009). Characteristics of bipolar disorder in an Australian specialist outpatient clinic: Comparison across large datasets. *Australian and New Zealand Journal of Psychiatry* **43**, 109-117.

**Mitchell, P. B., Johnston, A. K., Frankland, A., Slade, T., Green, M. J., Roberts, G., Wright, A., Corry, J. & Hadzi-Pavlovic, D.** (2013). Bipolar disorder in a national survey using the World Mental Health Version of the Composite International Diagnostic Interview: the impact of differing diagnostic algorithms. *Acta Psychiatrica Scandinavica* **127**, 381-93.

**Nakagawa, A., Grunebaum, M. F., Sullivan, G. M., Currier, D., Ellis, S. P., Burke, A. K., Brent, D. A., Mann, J. J. & Oquendo, M. A.** (2008). Comorbid anxiety in bipolar disorder: does it have an independent effect on suicidality? *Bipolar Disorders* **10**, 530-8.

**Negash, A., Alem, A., Kebede, D., Deyessa, N., Shibre, T. & Kullgren, G.** (2005). Prevalence and clinical characteristics of bipolar I disorder in Butajira, Ethiopia: a community-based study. *Journal of Affective Disorders* **87**, 193-201.

**Nery-Fernandes, F., Quarantini, L. C., Guimaraes, J. L., de Oliveira, I. R., Koenen, K. C., Kapczinski, F. & Miranda-Scippa, A.** (2012). Is there an association between suicide attempt and delay of initiation of mood stabilizers in bipolar I disorder? *Journal of Affective Disorders* **136**, 1082-1087.

**Neves, F. S., Malloy-Diniz, L. F. & Correa, H.** (2009). Suicidal Behavior in Bipolar Disorder: What Is the Influence of Psychiatric Comorbidities? *Journal of Clinical Psychiatry* **70**, 13-18.

**Parker, G., Fletcher, K., McCraw, S., Futeran, S. & Hong, M.** (2013). Identifying antecedent and illness course variables differentiating bipolar i, bipolar ii and unipolar disorders. *Journal of Affective Disorders* **148**, 202-209.

**Passos, I. C., Jansen, K., Cardoso, T. D., Colpo, G. D., Zeni, C. P., Quevedo, J., Kauer-Sant'Anna, M., Zunta-Soares, G., Soares, J. C. & Kapczinski, F.** (2016). Clinical Outcomes Associated With Comorbid Posttraumatic Stress Disorder Among Patients With Bipolar Disorder. *Journal of Clinical Psychiatry* **77**, E555-E560.

**Pawlak, J., Dmitrzak-Weglarz, M., Skibinska, M., Szczepankiewicz, A., Leszczynska-Rodziewicz, A., Rajewska-Rager, A., Maciukiewicz, M., Czerski, P. & Hauser, J.** (2013). Suicide attempts and psychological risk factors in patients with bipolar and unipolar affective disorder. *General Hospital Psychiatry* **35**, 309-313.

**Perugi, G., Micheli, C., Akiskal, H. S., Madaro, D., Socci, C., Quilici, C. & Musetti, L.** (2000). Polarity of the first episode, clinical characteristics, and course of manic depressive illness: a systematic retrospective investigation of 320 bipolar I patients. *Comprehensive Psychiatry* **41**, 13-8.

**Popovic, D., Vieta, E., Azorin, J. M., Angst, J., Bowden, C. L., Mosolov, S., Young, A. H. & Perugi, G.** (2015). Suicide attempts in major depressive episode: evidence from the BRIDGE-II-Mix study. *Bipolar Disorders* **17**, 795-803.

**Romero, S., Colom, F., Iosif, A. M., Cruz, N., Pacchiaroti, I., Sanchez-Moreno, J. & Vieta, E.** (2007). Relevance of family history of suicide in the long-term outcome of bipolar disorders. *Journal of Clinical Psychiatry* **68**, 1517-1521.

**Sanchez-Gistau, V., Colom, F., Mane, A., Romero, S., Sugranyes, G. & Vieta, E.** (2009). Atypical depression is associated with suicide attempt in bipolar disorder. *Acta Psychiatrica Scandinavica* **120**, 30-36.

**Sandberg, J. V., Jakobsson, J., Palsson, E., Landen, M. & Mathe, A. A.** (2014). Low neuropeptide Y in cerebrospinal fluid in bipolar patients is associated with previous and prospective suicide attempts. *European Neuropsychopharmacology* **24**, 1907-1915.

**Schaffer, A., Cairney, J., Veldhuizen, S., Kurdyak, P., Cheung, A. & Levitt, A.** (2010). A population-based analysis of distinguishers of bipolar disorder from major depressive disorder. *Journal of Affective Disorders* **125**, 103-110.

**Shabani, A., Teimurinejad, S., Kokar, S., Ahmadzad Asl, M., Shariati, B., Mousavi Behbahani, Z., Ghasemzadeh, M. R., Hasani, S., Taban, M., Shirekhoda, S., Ghorbani, Z., Tat, S., Nohesara, S. & Shariat, S. V.** (2013). Suicide Risk Factors in Iranian Patients With Bipolar Disorder: A 21- Month Follow-Up From BDPF Study. *Iranian Journal of Psychiatry and Behavioral Sciences* **7**, 16-23.

**Song, J. Y., Yu, H. Y., Kim, S. H., Hwang, S. S. H., Cho, H. S., Kim, Y. S., Ha, K. & Ahn, Y. M.** (2012). Assessment of Risk Factors Related to Suicide Attempts in Patients With Bipolar Disorder. *Journal of Nervous and Mental Disease* **200**, 978-984.

**Souery, D., Van Gestel, S., Massat, I., Blairy, S., Adolfsson, R., Blackwood, D., Del-Favero, J., Dikeos, D., Jakovljevic, M., Kaneva, R., Lattuada, E., Lerer, B., Lilli, L., Milanova, V., Muir, W., Nothen, M., Oruc, L., Papadimitriou, G., Propping, P., Schulze, T., Serretti, A., Shapira, B., Smeraldi, E., Stefanis, C., Thomson, M., Van Broeckhoven, C. & Mendlewicz, J.** (2001). Tryptophan hydroxylase polymorphism and suicidality in unipolar and bipolar affective disorders: A multicenter association study. *Biological Psychiatry* **49**, 405-409.

**Swann, A. C., Lijffijt, M., Lane, S. D., Steinberg, J. L. & Moeller, F. G.** (2009). Increased trait-like impulsivity and course of illness in bipolar disorder. *Bipolar Disorders* **11**, 280-8.

**Tamminga, C. A., Ivleva, E. I., Keshavan, M. S., Pearlson, G. D., Clementz, B. A., Witte, B., Morris, D. W., Bishop, J., Thaker, G. K. & Sweeney, J. A.** (2013). Clinical phenotypes of psychosis in the Bipolar-Schizophrenia Network on Intermediate Phenotypes (B-SNIP). *The American Journal of Psychiatry* **170**, 1263-1267.

**Tsai, S. Y., Lee, J. C. & Chen, C. C.** (1999). Characteristics and psychosocial problems of patients with bipolar disorder at high risk for suicide attempt. *Journal of Affective Disorders* **52**, 145-52.

**Tundo, A., Musetti, L., Benedetti, A., Berti, B., Massimetti, G. & Dell'Osso, L.** (2015). Onset polarity and illness course in bipolar I and II disorders: The predictive role of broadly defined mixed states. *Comprehensive Psychiatry* **63**, 15-21.

**Valtonen, H., Suominen, K., Mantere, O., Leppamaki, S., Arvilommi, P. & Isometsa, E. I.** (2005). Suicidal ideation and attempts in bipolar I and II disorders. *Journal of Clinical Psychiatry* **66**, 1456-1462.

**Zimmerman, M., Ellison, W., Morgan, T. A., Young, D., Chelminski, I. & Dalrymple, K.** (2015). Psychosocial morbidity associated with bipolar disorder and borderline personality disorder in psychiatric out-patients: comparative study. *British Journal of Psychiatry* **207**, 334-338.
